# Neuro-ophthalmologic symptoms after coronavirus disease 2019 vaccination: a retrospective study

**DOI:** 10.1186/s12886-022-02747-7

**Published:** 2023-01-05

**Authors:** Kyumin Kang, Se Youp Lee, Dong Cheol Lee

**Affiliations:** Department of Ophthalmology, School of Medicine, Keimyung University, 1095 Dalgubeol-Daero, Dalseo-Gu, Daegu, 42601 Republic of Korea

**Keywords:** Coronavirus, COVID-19, Ophthalmology, Vaccines

## Abstract

**Background:**

There have been several studies on inflammatory ophthalmic diseases; however, few studies have reported neuro-ophthalmological symptoms, such as diplopia and ocular motor nerve palsy, after coronavirus disease 2019 (COVID-19) vaccination. Therefore, this study aimed to report neuro-ophthalmological symptoms in patients after COVID-19 vaccination.

**Methods:**

This was a retrospective study based on the medical records of 10 patients who visited our ophthalmology clinic in 2021 with symptoms, such as diplopia (nine patients) and decreased visual acuity (one patient), and showed findings, such as ocular motor nerve palsy, after vaccination against COVID-19.

**Results:**

One patient had third nerve palsy, two had sixth nerve palsy, and five had fourth nerve palsy. One patient complained of subjective binocular diplopia but all test results were normal. One patient presented with decreased visual acuity accompanied by a sudden increase in intraocular pressure and orbital cellulitis in the other eye. The symptoms improved gradually in most patients. Compared with previous studies, this study reported three cases of antiplatelet therapy that was initiated due to the older age of the patients and underlying diseases.

**Conclusion:**

As COVID-19 vaccines can cause neuro-ophthalmological diseases, such as ocular motor nerve palsy, patients’ age and underlying diseases should be considered while administering them.

**Supplementary Information:**

The online version contains supplementary material available at 10.1186/s12886-022-02747-7.

## Background

On December 2, 2020, the United Kingdom approved the world's first emergency use of Pfizer's coronavirus disease 2019 (COVID-19) vaccine. The Republic of Korea started COVID-19 vaccination on February 26, 2021. The side effects of each vaccine vary despite the general symptoms of fever, myalgia, and headache. Thrombotic events after receiving the AstraZeneca (AZ) vaccine [1] and myocarditis and pericarditis associated with the Pfizer-BioNTech and Moderna vaccines have been reported [2].

Neurological and ophthalmic symptoms accompanying COVID-19 have been reported since the onset of the COVID-19 pandemic. In addition, inflammatory ophthalmic diseases after COVID-19 vaccination have been reported [3, 4]; however, neuro-ophthalmological symptoms are scarcely reported [5–10]. Therefore, this study aimed to report neuro-ophthalmological symptoms such as diplopia, decreased visual acuity, and signs of ocular motor nerve palsy in patients after COVID-19 vaccination.

## Methods

The medical records of 10 patients (nine with diplopia and one with decreased visual acuity) who visited our ophthalmology clinic from August 2021 to November 2021 with symptoms that developed after COVID-19 vaccination were reviewed. Data on the subjective symptoms, sex, age, medical history, visual acuity (VA) in decimal notation, alternate prism cover test (PCT), extraocular muscle movement (EOM) limitation, and Hess screen test were collected for all patients. None of the patients had contracted COVID-19 before vaccination. If necessary, a neurologist was consulted, and brain magnetic resonance imaging (MRI), cerebrospinal fluid studies, and blood tests were performed. The study adhered to the tenets of the Declaration of Helsinki for biomedical research involving humans. The Institutional Review Board of the Keimyung University Dongsan Medical Center (DSMC 2021–11-021) approved the study design and waived the need for obtaining informed consent due to the retrospective nature of this study. For patients who had their eye-movement photos taken, informed consent was obtained for the use of their images for research purposes and to publish the images in online open-access publications.

## Results

### Case 1

**Fig. 1:**
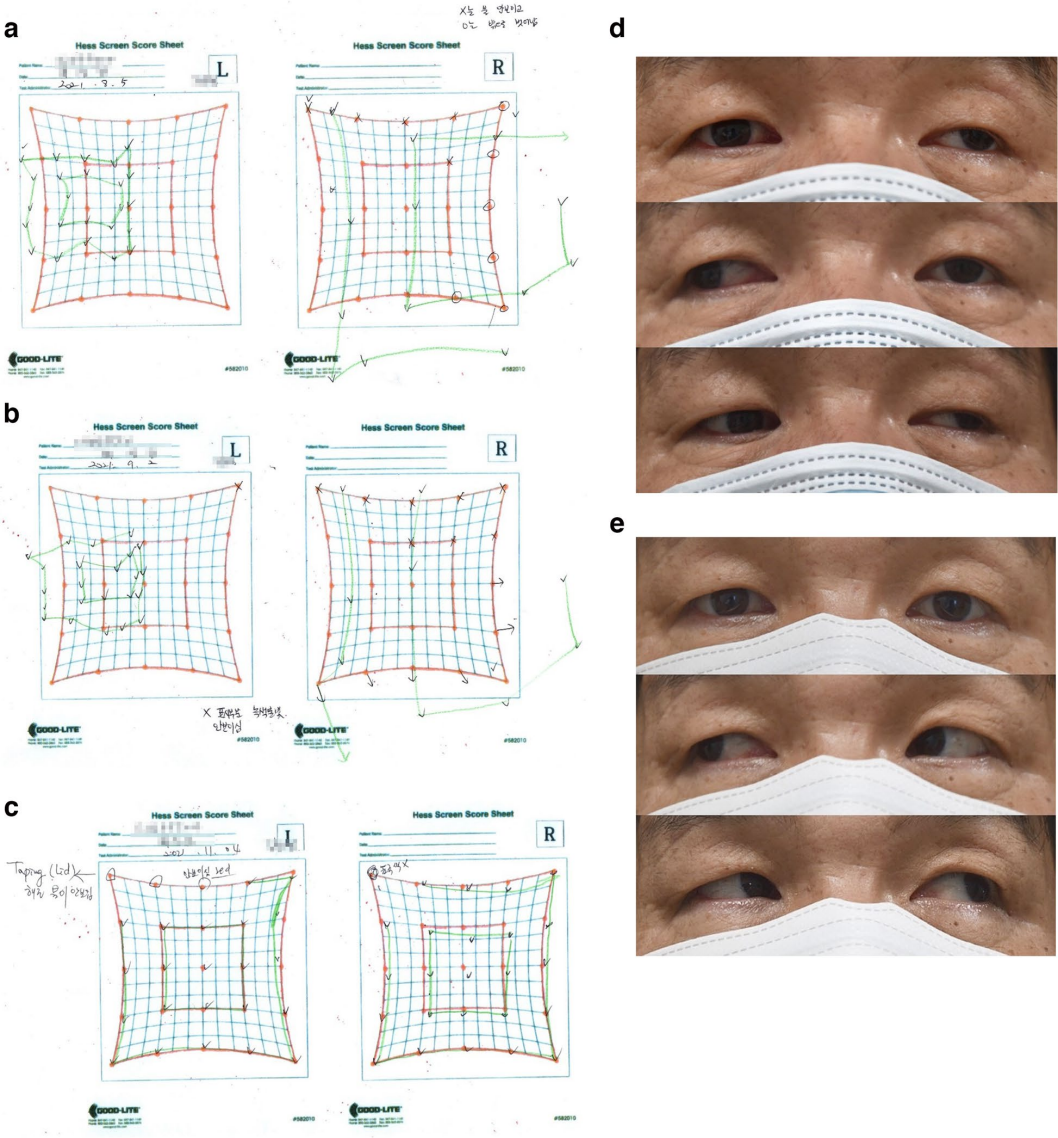
Hess screen tests and eye movement in case 1, demonstrating left third nerve palsy. **a** Hess screen test on day 4. **b** Hess screen test on day 13. **c** Hess screen test on week 6. **d** External photograph showing limitation −2 in all directions except lateral gaze in the OS. **e** External photograph showing full action in all directions

A 56-year-old man under medication for diabetes and hyperlipidemia and with a history of left facial nerve palsy visited our hospital on August 5, 2021, after experiencing binocular diplopia, left upper eyelid ptosis, and left frontal headache since the afternoon of the day of the first Pfizer vaccination on August 2, 2021. His best-corrected VA (BCVA) and intraocular pressure (IOP) were 1.0 and 14 mmHg, respectively, in oculus uterque (OU). There was EOM limitation in all directions except in the lateral gaze of the oculus sinister (OS) (Fig. 1d). The pupil sizes of OU were the same, and the light reflex was normal. There was exotropia of 25 prism diopters (PD) with oculus dextrus (OD) deviation on alternate PCT, and the Hess screen test (Fig. 1a) showed findings suggestive of left third nerve palsy. There were no specific findings for the cerebrospinal fluid study or brain MRI. Considering the possibility of an ischemic cause, aspirin 100 mg once a day was prescribed.

On August 17, 2021, the left ptosis slightly improved, but the EOM limitation did not. There was almost no change in the Hess screening test results (Fig. 1b). On the third visit on November 4, 2021, the left ptosis and EOM range had fully recovered (Fig. 1e), and no exotropia was observed on alternate PCT. The Hess screen test (Fig. 1c) showed full recovery to normal.

### Case 2

A 57-year-old woman on oral prednisolone for hypopituitarism after surgery for pituitary adenoma in 1997 developed binocular diplopia 2 weeks after the second dose of the Pfizer vaccine on September 11, 2021, and visited our hospital on October 29, 2021. Diplopia improved on tilting the head to the right. Her BCVA was 1.0 in OU, and the IOPs were 21 and 19 mmHg in the OD and OS, respectively. Left hypertropia of 5 PD was found on alternate PCT. Inferior oblique muscle overaction (IOOA) of 2 + was observed in the OS, and the Hess screen test showed left fourth nerve palsy (Figure S1a in Additional File 1). Brain MRI was performed on November 23, 2021, with no remarkable findings noted.

On December 15, 2021, the second visit, the IOOA in the OS decreased by + 1, and the left hypertropia decreased by 3 PD on alternate PCT, but the improvement was not complete. The Hess screen test also showed that the degree decreased, but diplopia persisted (Figure S1b in Additional File 1). On April 8, 2022, the third visit, the left hypertropia decreased by 1 PD on alternate PCT. The Hess screen test findings showed slight improvement (Figure S1c in Additional File 1), but diplopia still persisted. On July 8, 2022, the fourth visit, the Hess screen test revealed full recovery to normal status (Figure S1d), and the patient no longer complained of diplopia.

### Case 3

An 81-year-old woman with no remarkable medical history other than chronic non-tuberculous mycobacterial infection visited our outpatient clinic on July 12, 2021, after developing binocular vertical diplopia approximately a month after the second dose of the Pfizer vaccine on June 15, 2021. Her BCVAs of the OD and OS were 0.63 and 0.5, respectively, and the IOPs were 12 and 10 mmHg, respectively. There was an EOM limitation of −2 on lateral gaze in the OS; alternate PCT showed esotropia of 6 PD with OS deviation. Based on these findings and the Hess screen test result (Figure S2 in Additional File 1), the patient was diagnosed with left sixth nerve palsy. She was scheduled to revisit the clinic on September 8, 2021, but she did not come. We tried to contact her, but she was unreachable. Instead, she visited another department in our hospital in early December; the diplopia may have improved spontaneously.

### Case 4

A 70-year-old woman on hypertension medication for 15 years visited our outpatient clinic on November 27, 2021, because of binocular diplopia for 2 days, after receiving the first dose of the Pfizer vaccine on November 6, 2021. Diplopia was aggravated on the right gaze accompanied by a headache. Her BCVAs were 0.8 and 0.6 for the OD and OS, respectively, and the IOPs were 21 and 22 mmHg for the OD and OS, respectively. Alternate PCT showed esotropia of 14 PD at a far distance and 16 PD at a near distance with OD deviation. There was a limitation of abduction of −2 in the right eye, and the Hess screen test showed right sixth nerve palsy (Figure S3 in Additional File 1). She was scheduled to visit our clinic on December 3, 2021, but she did not. We contacted her on December 11, 2021, to inquire about her symptoms and were informed that her symptoms had improved. Brain MRI was performed at another hospital and no abnormal findings were noted.

### Case 5

A 67-year-old woman on medication for hypertension, hyperlipidemia, and hypothyroidism visited our neurology clinic on July 22, 2021, due to binocular diplopia that had occurred 3 days after receiving the first dose of the AZ vaccine in early June 2021. Her symptoms worsened when she was tired or watching television. In addition, her left eyelid drooped, and she said that it felt like her eyes were being pulled inward. She came to our hospital after undergoing a brain MRI at another hospital, which showed acute and old infarctions in the right basal ganglia, severe focal stenosis in the left M2 segment of the middle cerebral artery, and mild stenosis in both distal vertebral arteries. She had already been prescribed clopidogrel and cilostazol after MRI at that hospital. Repetitive nerve stimulation test and a serum anti-acetylcholine receptor antibody titer test were performed in consideration of myasthenia gravis in the neurology department, but no abnormal findings were revealed. She was referred to our eye clinic and had her first visit on September 24, 2021. Her BCVAs were 0.5 and 0.32 for the OD and OS, respectively, and the IOP for OU was 13 mmHg. Alternate PCT showed right hypertropia of 4 PD, and a 10° head tilt to the left was observed. She was diagnosed with right fourth nerve palsy based on these findings and the result of the Hess screen test (Figure S4a in Additional File 1). The results of the brain MRI performed in July did not correlate with her symptoms. Prism glasses with a power of 4 PD base down were prescribed for the OD.

At the follow-up visit on November 19, 2021, she reported that her left eyelid sagging had improved. The right hypertropia slightly improved to 3 PD from the previous value of 4 PD, but the right fourth nerve palsy showed little improvement on the Hess screen test (Figure S4b in Additional File 1). On November 25, 2021, she received the Pfizer vaccine instead of AZ as the second dose of the COVID-19 vaccine. On November 27, 2021, she returned to the outpatient clinic and reported that her glasses did not fit well and that she felt dizzy after the second vaccination; there was almost no change in the Hess screen test (Figure S4c in Additional File 1). She was provided with a new prescription; the powers of the prism glasses were corrected from 4 to 3 PD base down.

### Case 6

**Fig. 2:**
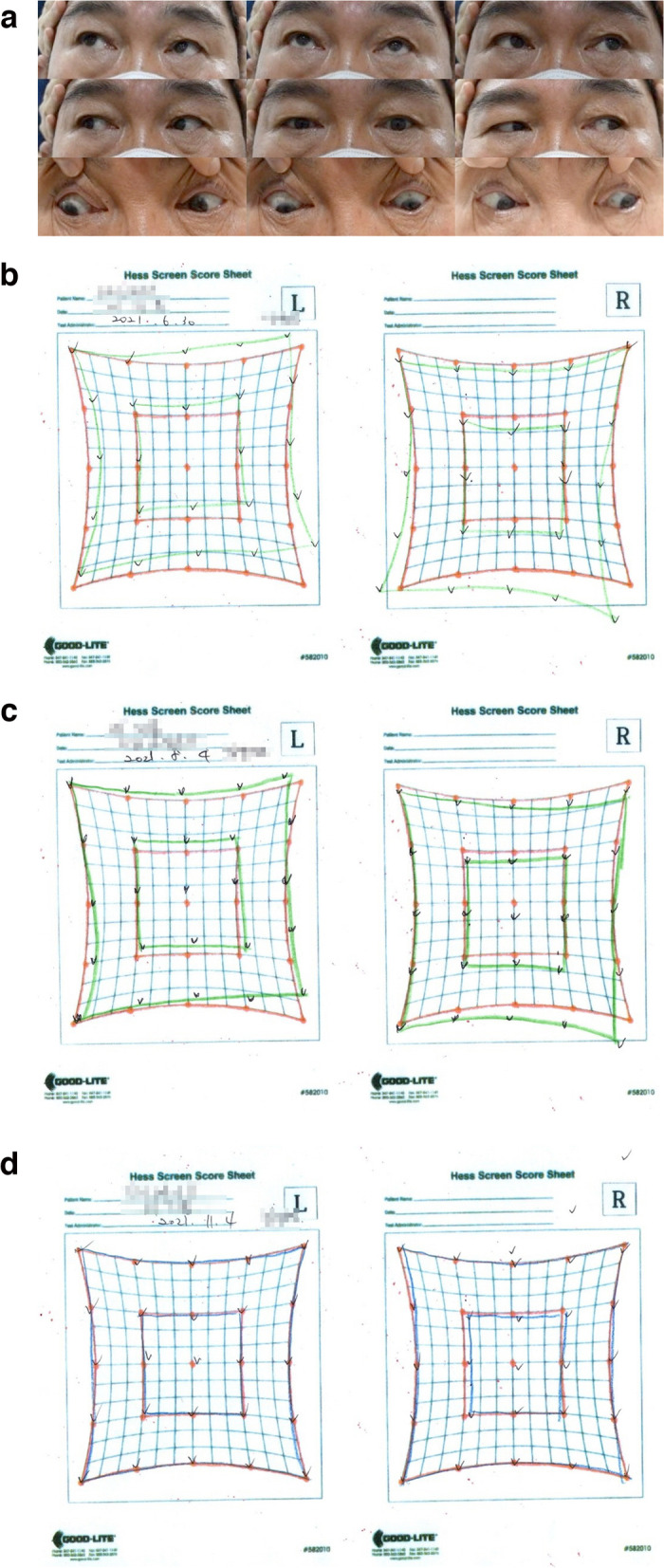
Hess screen tests and eye movements in case 6, demonstrating left fourth nerve palsy. **a** Nine cardinal gaze directions showing left superior oblique underaction on day 3. **b** Hess screen test on day 15. **c** Hess screen test on week 7. **d** Hess screen test on week 20

A 60-year-old man on oral medication for diabetes, hypertension, and hyperlipidemia, including clopidogrel, underwent demarcation laser treatment for retinal holes of OU in 2010. He received the first dose of AZ on June 14, 2021. Two days later, he developed an occipital headache and binocular horizontal diplopia when looking straight ahead or to the right. He was admitted to the emergency room on June 18, 2021. His BCVAs were 0.8 both for the OD and OS, and his IOPs were 13 mmHg both for the OD and OS. There was no EOM limitation, but left superior oblique underaction (SOUA) was present (Fig. 2a). He had exotropia of 4 PD with OS deviation in the primary position, right gaze, and left gaze. Left hypertropia of 4 PD occurred only when he looked to the right. His head was tilted to the right at rest, and the diplopia was aggravated when he tilted his head to the left. A right distal internal carotid artery aneurysm was observed on brain MRI, which was an incidental finding that did not correlate with the symptoms. The patient was considered to have left fourth nerve palsy, and he continued taking clopidogrel and undergoing outpatient follow-up.

On June 30, 2021, during a follow-up visit, he had an IOOA of 1 + in the OS and left hypertropia of 4 PD. The left fourth nerve palsy persisted on the Hess screen testing (Fig. 2b). At the second follow-up visit on August 4, 2021, the degree of hypertropia had reduced to 1 PD, and the diplopia had improved based on the Hess screen testing (Fig. 2c).

At the last follow-up visit on November 4, 2021, 5 months after the onset of diplopia, the symptoms had almost resolved. There was no IOOA, exotropia, or hypertropia, and the Hess screen test result was also normal (Fig. 2d).

### Case 7

A 69-year-old man on oral medication for hyperlipidemia and with a history of cataract surgery in OU in 2015 was vaccinated with the first dose of AZ on June 2, 2021. Twelve days later, he suddenly developed binocular diagonal diplopia, which warranted him to visit our outpatient clinic. His uncorrected VAs were 1.0 and 1.0 for the OD and OS, respectively, and the IOPs were 11 and 12 mmHg for the OD and OS, respectively. A left head tilt of 5° was observed, and the alternate PCT showed right hypertropia of 6 PD. Based on these findings and the Hess screen test results (Figure S5a in Additional File 1), right fourth nerve palsy was diagnosed. No acute infarction was observed, but mild-to-moderate small vessel disease in the cerebral white matter with a few old lacunar infarcts and old microbleeds were found on the brain MRI, which did not correlate with the symptoms. Aspirin was prescribed as prophylaxis. At the follow-up visit on July 12, 2021, the patient reported that the diplopia had resolved 1 day after symptom onset. The Hess screen test results were normal (Figure S5b in Additional File 1), even without performing head tilt. No strabismus was observed.

### Case 8

**Fig. 3:**
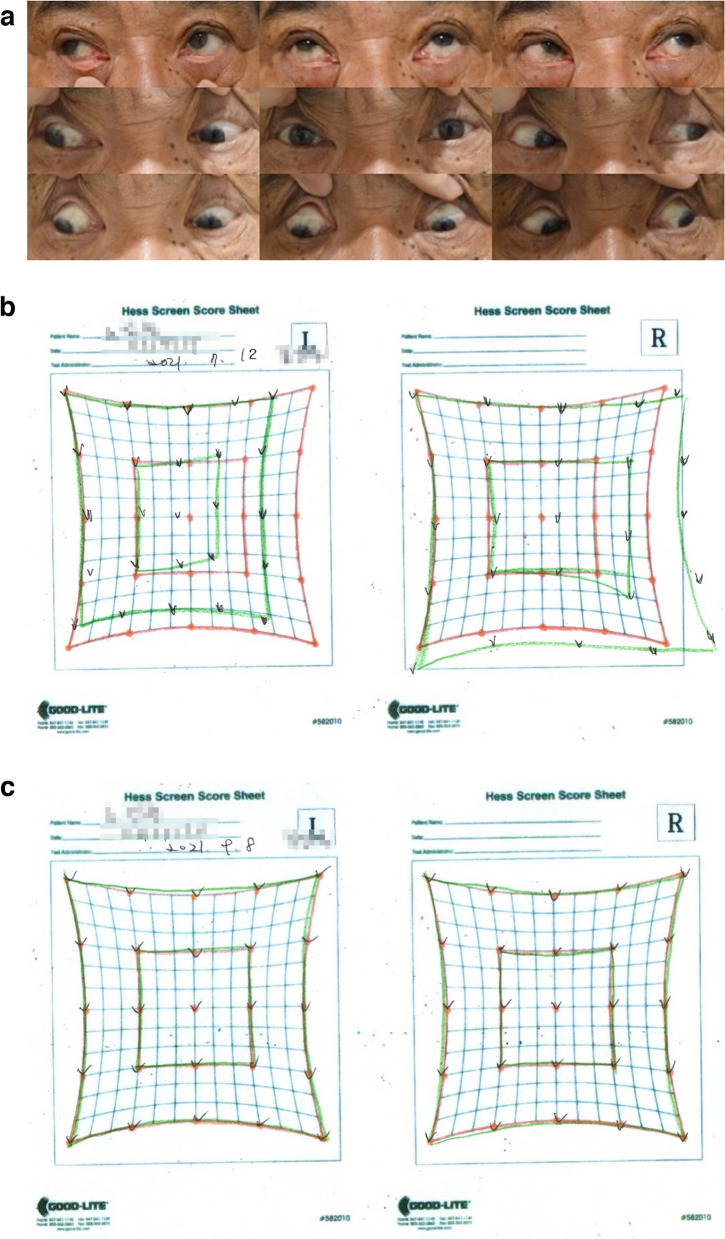
Hess screen test and eye movement in case 8, demonstrating left fourth nerve palsy. **a** Nine cardinal gaze directions showing EOM limitation 0.5− with medial and inferior gaze, inferior oblique overaction 0.5 + , and superior oblique underaction 0.5 + in the OS on day 3. **b** Hess screen test on day 6. **c** Hess screen test on week 9

A 68-year-old man with a medical history of hypertension, old cerebral infarction, rheumatoid arthritis, and migraine developed binocular vertical diplopia on July 7, 2021, after receiving the first dose of the AZ vaccine on June 14, 2021. He was admitted to the emergency room on July 10, 2021, due to dizziness and right facial numbness. His BCVAs were 1.0 and 0.8 for the OD and OS, respectively, and the IOPs were 16 and 15 mmHg for the OD and OS, respectively. There was an EOM limitation of 0.5 on the medial and inferior gaze directions for the OS. An IOOA of 0.5 + and a SOUA of 0.5 + in the OS were observed (Fig. 3a). He had left exotropia of 8 PD with left hypertropia of 4 PD when looking straight ahead. The deviation worsened to exotropia of 18 PD with left hypertropia of 6 PD on right gaze and improved to no exotropia with left hypertropia of 3 PD on left gaze. Left fourth nerve palsy was diagnosed based on these findings.

Blood tests showed no specific findings other than a slight increase in erythrocyte sedimentation rate (ESR) to 17 mm/h and an increase in triglycerides of 308 mg/dL. The brain MRI showed a small acute infarct with restricted diffusion in the right occipital lobe, mild small vessel disease in the cerebral white matter, and severe segmental stenosis in the right distal vertebral artery. He was admitted by a neurologist for close observation. Two days later, left hypertropia of 5 PD, right head tilt of 10°, and an IOOA of 1 + in the OS were observed, and the Hess screen test showed left fourth nerve palsy (Fig. 3b). Clopidogrel was added to supplement aspirin, and statin therapy was started considering the possibility of a vascular cause. On July 13, 2021, 3 days after the onset of symptoms, the patient reported that the diplopia had resolved. He was discharged on July 16, 2021.

At the follow-up visit on September 8, 2021, no strabismus-related findings on alternate PCT were found, and the Hess screen test showed normal findings (Fig. 3c).

### Case 9

A 65-year-old woman with no significant medical history visited our hospital on July 5, 2021, due to decreased VA and binocular diplopia after receiving the first dose of the AZ vaccine (June 8, 2021). The diplopia worsened on the right gaze. The BCVAs were 1.0 and 1.0 for the OD and OS, respectively, and the IOP was 17 mmHg for OU. There was exotropia of 4 PD at far on alternate PCT, and the Hess screen test result was normal (Figure S6a in Additional File 1). The findings on slit-lamp examination, fundoscopy, and optical coherence tomography (OCT) were unremarkable. On July 19, 2021, she reported that her symptoms had improved; examination results were the same as those at the first visit (Figure S6b in Additional File 1).

### Case 10

A 73-year-old woman was vaccinated with the first dose of the Moderna vaccine on September 16, 2021, and in the afternoon, she experienced vision loss in the OD, severe discharge in the OS, and left temporal headache. She was admitted to the emergency room on September 17, 2021. She had accompanying chills, myalgia, cough, and rhinorrhea. In 2019, the VA in the OD was 0.63; however, the VA decreased significantly to hand motion during this visit.

The OD IOP was elevated (31 mmHg), but the angle was open. Edema or other abnormalities were not noted in the cornea, except for mild superficial punctate keratitis, and no prominent retinal or vitreous lesions were observed on fundoscopy and OCT. These were not correlated with a sudden decrease in VA. Brain MRI was performed to rule out brain-related causes; however, apart from an incidentally found aneurysm at the bifurcation of the right middle cerebral artery, no lesions that could cause visual loss were observed, and only left eyelid edema with contrast enhancement was a remarkable finding. Considering the possibility that the visual loss in the OD may be due to an increase in IOP, intravenous mannitol (75 g) injection was administered. After the injection, IOP decreased to 11 mmHg, and VA improved to 0.2. Instillations of dorzolamide/timolol (Cosopt), brimonidine (Alphagan P), and latanoprost (Xalatan) in the OD were initiated.

Regarding the discharge from the OS, blood tests revealed leukocytosis and a C-reactive protein (CRP) concentration level of 2.5 mg/dL, and ESR increased to 87 mm/h. Based on these findings, including the MRI findings, the possibility of orbital cellulitis was considered. After hospitalization, intravenous ampicillin/sulbactam (3 g) was administered four times a day every 6 h, and methylprednisolone 250 mg was administered once a day for 5 days. For the OS, levofloxacin (Cravit) 1.5% eye solution four times daily, ofloxacin (Ocuflox) eye ointment four times daily, and wiping the eyelid with lid scrub cleanser (Ocusoft) were prescribed for 5 days.

The OS swelling gradually improved, and the uncorrected VA of the OD also improved to 0.63^–1^. CRP and ESR decreased to 0.7 mg/dL and 94 mm/h, respectively. No bacteria were identified from the blood culture and eye discharge culture tests. The patient was discharged on the fifth day of hospitalization. During the follow-up on October 1, 2021, the findings were maintained without exacerbation.

## Discussion

In this study, among the nine patients who developed diplopia, four (cases 1, 6, 7, and 8) showed total resolution of the subjective symptoms confirmed via examination and one (case 4) reported total resolution of the subjective symptoms without confirmation. One patient (case 9) complaining of binocular diplopia showed normal test results compared with those from the first visit, although the symptoms improved spontaneously during the follow-up. Two patients (cases 2 and 5) had residual hypertropia with improved symptoms, which persisted at the most recent follow-up. One patient was lost to follow-up after the first visit, but the condition is presumed to have improved (Table 1). In this study, all 10 patients were older than 50 years (minimum, 56 years; maximum, 81 years). In other studies [5–10], only four of eight patients were older than 50 years. The patients in this study were older than those in other reports [5–10]. Regarding the types of COVID-19 vaccine, four cases with Pfizer, five cases with AZ, and one case with Moderna vaccines were reported in this study. In previous studies [5–10], three cases with Pfizer [6, 8, 9], one case with AZ [10], one case with Moderna [5], and three cases with recombinant adenovirus vector encoding spike (S) glycoprotein vaccines (unknown company name) [7] were reported. Table 2 summarizes six previous reports [5–10] on eight patients with ocular motor nerve palsies following COVID-19 vaccination. In summary, one patient had right oculomotor nerve palsy with anti-GQ1b Ab [8], and another had multiple nerve palsy (left oculomotor, abducens, trigeminal, and facial palsies) [9]. Among the remaining patients, there were four cases of abducens nerve palsy [6, 7, 10], one of oculomotor nerve palsy [5], and one of bilateral vertical gaze palsy [7].

**Table 1:** Summary of this case series

Case	Age (years)	Sex	Vaccine	Days after vaccine	Symptoms	Manifestations	Treatment and outcome
1	56	M	Pfizer-BioNTech #1	0	Binocular diplopia, left ptosis, left frontal headache	Left third nerve palsy	Total resolution within 3 months (aspirin started)
2	57	F	Pfizer-BioNTech #2	14	Binocular diplopia	Left fourth nerve palsy	Residual left hypertropia of 3 PD (symptoms persisted for > 3 months)
3	81	F	Pfizer-BioNTech #2	28	Binocular vertical diplopia	Left sixth nerve palsy	Follow-up loss (presumed to have had spontaneous resolution)
4	70	F	Pfizer-BioNTech #1	19	Binocular diplopia, headache	Right sixth nerve palsy	Spontaneous total resolution of symptoms within 18 days (relied on patient’s statements, no tests were performed)
5	67	F	AZ #1	3	Binocular diplopia, left ptosis	Right fourth nerve palsy	Residual right hypertropia of 3 PD, prism glasses prescribed (symptoms persisted for > 6 months)
6	60	M	AZ #1	2	Binocular horizontal diplopia, occipital headache	Left fourth nerve palsy	Spontaneous total resolution within 5 months
7	69	M	AZ #1	12	Binocular diagonal diplopia	Right fourth nerve palsy	Total resolution within 1 day (aspirin started)
8	68	M	AZ #1	23	Binocular vertical diplopia, dizziness, right facial numbness	Left fourth nerve palsy	Total resolution within 6 days (dual antiplatelet therapy started)
9	65	F	AZ #1	3	Binocular diplopia (subjective)	Diplopia due to undetermined cause	Spontaneous recovery within 6 weeks
10	73	F	Moderna #1	0	Sudden loss of vision in the OD, severe discharge in the OS, left temporal headache, myalgia, dizziness, nausea, cough, rhinorrhea	Sudden loss of vision with increased IOP and orbital cellulitis	Recovery of vision after drop in IOP in 1 day, resolution of orbital cellulitis after intravenous antibiotics and corticosteroid for 5 days

*M* Male, *F* Female, *#1* First dose, *#2* Second dose, *OD* Oculus dexter, *OS* Oculus sinister, *AZ* AstraZeneca, *IOP* Intraocular pressure, *PD* Prism diopters

**Table 2:** Summary of other case reports demonstrating ocular motor nerve palsy after COVID-19 vaccination

Reports	Age (Years)	Sex	VaccinE	Days after vaccine	ManifestationS	Treatment and outcome
Pappaterra et al. [5]	81	M	Moderna #1	1	Left partial oculomotor nerve palsy	Spontaneous near-total resolution within 2 weeks
Reyes-Capo et al. [6]	59	F	Pfizer-BioNTech #1	2	Right abducens nerve palsy	Sensorimotor exam remained unchanged at the most recent follow-up
Pawar et al. [7]	24	F	^a^	21	Both vertical gaze palsy	Complete resolution within 10 days of systemic steroids
Pawar et al. [7]	44	M	a	30	Left abducens nerve palsy	Minimal residual esotropia after administering OS botulinum toxin injection to the medial rectus
Pawar et al. [7]	"Young"	M	a	6	Left abducens nerve palsy	Spontaneous resolution within 4 weeks
Kubota et al. [8]	65	M	Pfizer-BioNTech #2	17	Right oculomotor nerve palsy with anti-GQ1b Ab	Resolution after intravenous immunoglobulin therapy for 5 days
Manea et al. [9]	29	M	Pfizer-BioNTech #1	6	Multiple nerve palsy (left oculomotor, abducens, trigeminal, and facial nerve palsies)	Minimal left facial palsy after intravenous corticosteroid for 5 days
Pereira and Haslett [10]	65	M	AZ #2	3	Right abducens nerve palsy	Spontaneous total resolution within 3 months

*M* Male, *F* Female, *#1* First dose, *#2* Second dose, *OS* Oculus sinister, *AZ* AstraZeneca

^a^Recombinant, adenovirus vector encoding the SARS-CoV-2 spike (S) glycoprotein vaccine

In this study, seven of nine patients showed improvement in diplopia with a period from symptom onset to total resolution varying from 1 day to 5 months. In two cases (cases 2 and 5), 3 and 6 months had elapsed since the onset of symptoms, but there was no complete improvement. In other reports [5–10], seven of eight patients showed near-total to total resolution [5, 7–10], and the period from symptom onset to resolution ranged from 5 days to 3 months; none of the patients in these reports was prescribed antiplatelet drugs. However, in the present report, antiplatelet therapy was started for three of eight patients with ocular motor nerve palsy (cases 1, 7, and 8), and the most likely causes were vascular. One patient (case 6) who was previously taking clopidogrel continued taking it and did not receive any other treatment. All four patients had underlying conditions, such as diabetes, hypertension, hyperlipidemia, and cerebral infarction. This difference between the present study and other studies could be due to the age and underlying disease(s) of the patients. Ocular motor nerve palsy is a significant risk factor for subsequent stroke, and the risk of stroke continues up to 12 years [11–14]; therefore, to prevent future strokes, it is advisable to start antiplatelet therapy for cases of ocular motor nerve palsy with an underlying predisposition.

We believe that the diplopia in the above cases was caused by the COVID-19 vaccine because symptoms occurred within a month from the day of vaccination with no other specific events. In addition, most possible causes, including stroke, aneurysm, and infection, were excluded based on the brain MRI and blood test results. For case 8, there was an underlying predisposition to cerebral infarction history, and this event was also considered by a neurologist to be due to ischemic causes. AZ vaccination may have caused the ischemic trochlear nerve palsy by forming blood clots.

Several cases of binocular diplopia due to ocular motor nerve palsy after contracting COVID-19 have been reported [15–22]. Considering these cases, ocular motor nerve palsy after COVID-19 vaccination may be due to an immune response similar to that of COVID-19. Although rare, third, fourth, and sixth nerve palsies after routine vaccinations, including those for influenza and *Hemophilus influenzae* type b, have been reported [23]. Therefore, these responses may be related to COVID-19 or its vaccines, which may have evoked an immune response.

For case 9, the patient complained of binocular diplopia and metamorphopsia on testing with M-charts, but there were no signs of inflammation or other abnormalities on slit examination, fundoscopy, Hess screen test, or visual field test. The possible causes include the sudden discovery of pre-existing symptoms, convergence and divergence insufficiency, and the possibility of temporary aggravation of dry eye.

In case 10, orbital cellulitis developed in the OS after COVID-19 vaccination and improved after intravenous antibiotics and corticosteroid therapy. Rather than the COVID-19 vaccine itself causing cellulitis directly, the vaccine may have contributed to the exacerbation of inflammation, considering the rapid aggravation of systemic symptoms, such as myalgia, cough, and rhinorrhea, after vaccination. Orbital cellulitis has been reported to occur after COVID-19 [24, 25]. Cases of ocular inflammatory reactions, such as uveitis after COVID-19 vaccination [25–29], have also been reported, but there have been no reports of orbital cellulitis after COVID-19 vaccination. To the best of our knowledge, this is the first study to report orbital cellulitis following COVID-19 vaccination. Previously, approximately 11 cases of myositis or orbital inflammation occurring due to a routine vaccination other than that for COVID-19 were reported to the Vaccine Adverse Event Reporting System between 2010 and 2020 [30]. However, this patient was special because she had an underlying predisposition to systemic inflammation. She had previously been diagnosed with seropositive rheumatoid arthritis and was placed on methotrexate and tacrolimus, but she stopped taking medicine after symptom onset. CRP concentration and ESR had increased again without ophthalmic symptoms during the outpatient visit to the rheumatology department on December 1. Thus, she had a tendency to develop systemic inflammation, and the COVID-19 vaccine is believed to have triggered its exacerbation. In addition, there were no reported cases of a sudden increase in IOP or decrease in visual acuity of up-to-hand motion with recovery of visual acuity after lowering IOP after COVID-19 vaccination, as in case 10. However, she was lost to follow-up after 2019, and the baseline IOP before the onset of symptoms was unknown. After this event, the VA in the OD decreased to 0.1 again at the outpatient follow-up visit on October 1. In this regard, the accuracy of this patient’s visual acuity test may have been somewhat low: she may have had a pre-existing low visual acuity that was incidentally discovered after vaccination. Nevertheless, when the IOP increased after vaccination, VA dropped to hand motion; the VA was 0.1 or higher after the IOP decreased, and it increased with time during the hospitalization (0.1 → 0.12 → 0.16 → 0.5 → 0.63). The VA may have deteriorated after vaccination. The mechanism of increased IOP and decreased VA in this patient is still unclear.

This study has some limitations. First, it was a retrospective study. Second, although ocular motor nerve palsies occurred after COVID-19 vaccination, the underlying causal relationship and mechanisms are unclear. The patients characteristically complained of diplopia after COVID-19 vaccination, and no infarction was observed in the brain images of 4 (cases 1, 2, 4, and 6) of 7 (cases 1, 2, 4, 5, 6, 7, 8) patients who underwent brain work up; therefore, no other possible causes were considered. We determined that an association between ocular motor nerve palsies and the COVID-19 vaccination could not be ruled out. Third, if it was found that the patients had no other causes, it would have been favorable to consider myasthenia gravis. However, no evidence of ptosis or intraday fluctuations, which are common in ocular myasthenia gravis, was noted in most patients; therefore, tests for myasthenia gravis diagnosis (e.g., Tensilon test, repetitive nerve stimulation test, and serum anti-acetylcholine receptor antibody titer) were only performed for one patient (case 5). As the symptoms resolved spontaneously without treatment for myasthenia gravis, the possibility that the cause of diplopia was myasthenia gravis seems less likely at this time. Fourth, the vaccines received by the patients varied, and the interval between the visits was not constant. To elucidate a clear association between ocular motor nerve palsy and the COVID-19 vaccination, further evaluation with more cases is needed. However, compared with other studies, our study mainly showed eye movement disorders after each vaccination and direct results of certain tests (Hess screen and eyeball movement picture). In addition, our study showed that older adults had more underlying conditions and required antiplatelet treatment, which has not been reported in other studies.

## Conclusions

Our results suggest that ocular motor nerve palsy may occur as an adverse event of COVID-19 vaccines. Therefore, for patients with binocular diplopia after COVID-19 vaccination, but without other obvious causes, the side effects of vaccination should be considered. It often improves spontaneously over time, and follow-up or symptomatic treatment (e.g., prism glasses), depending on the clinical course of the patients, may be attempted. In addition, considering the thrombus formation tendency of COVID-19 vaccines, such as AZ, it would be beneficial to consider antiplatelet therapy for future prognosis and prevention of stroke, especially if there are underlying diseases such as diabetes, hypertension, and hyperlipidemia [11–14].

## Supplementary Information

**Additional file 1. Figure S1.** Hess screen tests in case 2, demonstrating left fourth nerve palsy. Hess screen test shows left fourth nerve palsy. (a) Week 5. (b) Week 12. (c) Week 28. (d) Week 41. **Figure S2.** Hess screen test in case 3, demonstrating left sixth nerve palsy. Hess screen test shows left sixth nerve palsy. **Figure S3.** Hess screen test in case 4, demonstrating right sixth nerve palsy. Hess screen test shows right sixth nerve palsy. **Figure S4.** Hess screen tests in case 5, demonstrating right fourth nerve palsy. Hess screen test shows right fourth nerve palsy. (a) Week 16. (b) Week 25. (c) Week 27. **Figure S5.** Hess screen test in case 7, demonstrating right fourth nerve palsy. Hess screen test shows right fourth nerve palsy. (a) Day 1. (b) Week 4. **Figure S6.** Hess screen test in case 9, demonstrating no eye movement defect. Hess screen test shows no eye movement defect. (a) Day 25. (b) Week 5.

## Data Availability

All data generated or analyzed during this study are included in this published article and its supplementary information files.

## Abbreviations

AZ: AstraZeneca
BCVA: Best-corrected visual acuity
COVID-19: Coronavirus disease 2019
EOM: Extraocular muscle movement
ESR: Erythrocyte sedimentation rate
IOOA: Inferior oblique muscle overaction
IOP: Intraocular pressure
MRI: Magnetic resonance imaging
OD: Oculus dextrus
OS: Oculus sinister
OU: Oculus uterque
PCT: Prism cover test
PD: Prism diopters
SOUA: Superior oblique underaction
VA: Visual acuity

## References

[1] European Medicines Agency. AstraZeneca’s COVID-19 vaccine: EMA finds possible link to very rare cases of unusual blood clots with low blood platelets. https://www.ema.europa.eu/en/news/astrazenecas-covid-19-vaccine-ema-finds-possible-link-very-rare-cases-unusual-blood-clots-low-blood. Accessed 19 Dec 2021.

[2] Shay DK, Shimabukuro TT, DeStefano F (2021). Myocarditis occurring after immunization with mRNA-based COVID-19 vaccines. JAMA Cardiol.

[3] Eleiwa TK, Gaier ED, Haseeb A, ElSheikh RH, Sallam AB, Elhusseiny AM (2021). Adverse ocular events following COVID-19 vaccination. Inflamm Res.

[4] Ng XL, Betzler BK, Testi I, Ho SL, Tien M, Ngo WK (2021). Ocular adverse events after COVID-19 vaccination. Ocul Immunol Inflammm.

[5] Pappaterra MC, Rivera EJ, Oliver AL (2021). Transient oculomotor palsy following the administration of the messenger RNA-1273 vaccine for SARS-CoV-2 diplopia following the COVID-19 vaccine. J Neuroophthalmol.

[6] Reyes-Capo DP, Stevens SM, Cavuoto KM (2021). Acute abducens nerve palsy following COVID-19 vaccination. J AAPOS.

[7] Pawar N, Maheshwari D, Ravindran M, Padmavathy S (2021). Ophthalmic complications of COVID-19 vaccination. Indian J Ophthalmol.

[8] Kubota T, Hasegawa T, Ikeda K, Aoki M (2021). Case report: isolated, unilateral oculomotor palsy with anti-GQ1b antibody following COVID-19 vaccination. F1000Res.

[9] Manea MM, Dragoș D, Enache I, Sirbu AG, Tuta S (2022). Multiple cranial nerve palsies following COVID-19 vaccination—case report. Acta Neurol Scand.

[10] Pereira A, Haslett RS (2021). Acute abducens nerve palsy following the second dose of the AstraZeneca COVID-19 vaccine. J Pediatr Ophthalmol Strabismus.

[11] Park SJ, Yang HK, Byun SJ, Park KH, Hwang JM (2018). Ocular motor cranial nerve palsy and increased risk of stroke in the general population. PLoS ONE.

[12] Galtrey CM, Schon F, Nitkunan A (2015). Microvascular non-arteritic ocular motor nerve palsies—what we know and how should we treat?. Neuroophthalmology.

[13] Rim TH, Han J, Choi YS, Lee T, Kim SS (2017). stroke risk among adult patients with third, fourth or sixth cranial nerve palsy: a nationwide cohort study. Acta Ophthalmol.

[14] Shew W, Wang MTM, Danesh-Meyer HV (2022). Stroke risk after ocular cranial nerve palsy—a systematic review and meta-analysis. J Clin Neurosci.

[15] Francis JE (2021). Abducens palsy and anosmia associated with COVID-19: a case report. Br Ir Orthopt J.

[16] Falcone MM, Rong AJ, Salazar H, Redick DW, Falcone S, Cavuoto KM (2020). Acute abducens nerve palsy in a patient with the novel coronavirus disease (COVID-19). J AAPOS.

[17] Dinkin M, Gao V, Kahan J, Bobker S, Simonetto M, Wechsler P (2020). COVID-19 presenting with ophthalmoparesis from cranial nerve palsy. Neurology.

[18] Lonardi V, Meneghesso D, Debertolis G, Pin JN, Nosadini M, Sartori S (2021). Isolated third cranial nerve palsy and COVID-19 infection in a child. Pediatr Neurol.

[19] de Oliveira MR, Lucena ARVP, Higino TMM, Ventura CV (2021). Oculomotor nerve palsy in an asymptomatic child with COVID-19. J AAPOS.

[20] Douedi S, Naser H, Mazahir U, Hamad AI, Sedarous M (2021). Third cranial nerve palsy due to COVID-19 infection. Cureus.

[21] Elenga N, Martin E, Gerard M, Osei L, Rasouly N (2021). Unilateral diplopia and ptosis in a child with COVID-19 revealing third cranial nerve palsy. J Infect Public Health.

[22] Faucher A, Rey PA, Aguadisch E, Degos B (2020). Isolated post SARS-CoV-2 diplopia. J Neurol.

[23] Woo EJ, Winiecki SK, Ou AC (2014). Motor palsies of cranial nerves (excluding VII) after vaccination: reports to the US vaccine adverse event reporting system. Hum Vaccin Immunother.

[24] Turbin RE, Wawrzusin PJ, Sakla NM, Traba CM, Wong KG, Mirani N (2020). Orbital cellulitis, sinusitis and intracranial abnormalities in two adolescents with COVID-19. Orbit.

[25] Carvalho VA, Vergínio VEO, Brito GC, Pereira-Stabile CL, Stabile GAV (2021). Coronavirus disease 2019 as a possible cause of severe orbital cellulitis. J Craniofac Surg.

[26] ElSheikh RH, Haseeb A, Eleiwa TK, Elhusseiny AM (2021). Acute uveitis following COVID-19 vaccination. Ocul Immunol Inflammm.

[27] Goyal M, Murthy SI, Annum S (2021). Bilateral multifocal choroiditis following COVID-19 vaccination. Ocul Immunol Inflammm.

[28] Mudie LI, Zick JD, Dacey MS, Palestine AG (2021). Panuveitis following vaccination for COVID-19. Ocul Immunol Inflammm.

[29] Renisi G, Lombardi A, Stanzione M, Invernizzi A, Bandera A, Gori A (2021). Anterior uveitis onset after bnt162b2 vaccination: is this just a coincidence?. Int J Infect Dis.

[30] Cheng JY, Margo CE (2022). Ocular adverse events following vaccination: overview and update. Surv Ophthalmol.

