# Supplementary material for: Neuro-ophthalmologic symptoms after coronavirus disease 2019 vaccination: a retrospective study
Source: BMC Ophthalmol. 2023 Jan 5;23:11. doi: 10.1186/s12886-022-02747-7 (PMC9813886; doi:10.1186/s12886-022-02747-7)
Supplement: Supplementary file 1 — Additional file 1. Figure S1. Hess screen tests in case 2, demonstrating left fourth nerve palsy. Hess screen test shows left fourth nerve palsy. (a) Week 5. (b) Week 12. (c) Week 28. (d) Week 41. Figure S2. Hess screen test in case 3, demonstrating left sixth nerve palsy. Hess screen test shows left sixth nerve palsy. Figure S3. Hess screen test in case 4, demonstrating right sixth nerve palsy. Hess screen test shows right sixth nerve palsy. Figure S4. Hess screen tests in case 5, demonstrating right fourth nerve palsy. Hess screen test shows right fourth nerve palsy. (a) Week 16. (b) Week 25. (c) Week 27. Figure S5. Hess screen test in case 7, demonstrating right fourth nerve palsy. Hess screen test shows right fourth nerve palsy. (a) Day 1. (b) Week 4. Figure S6. Hess screen test in case 9, demonstrating no eye movement defect. Hess screen test shows no eye movement defect. (a) Day 25. (b) Week 5. [file 12886_2022_2747_MOESM1_ESM.docx]

**Additional File 1**

**File format**: Word (.docx)


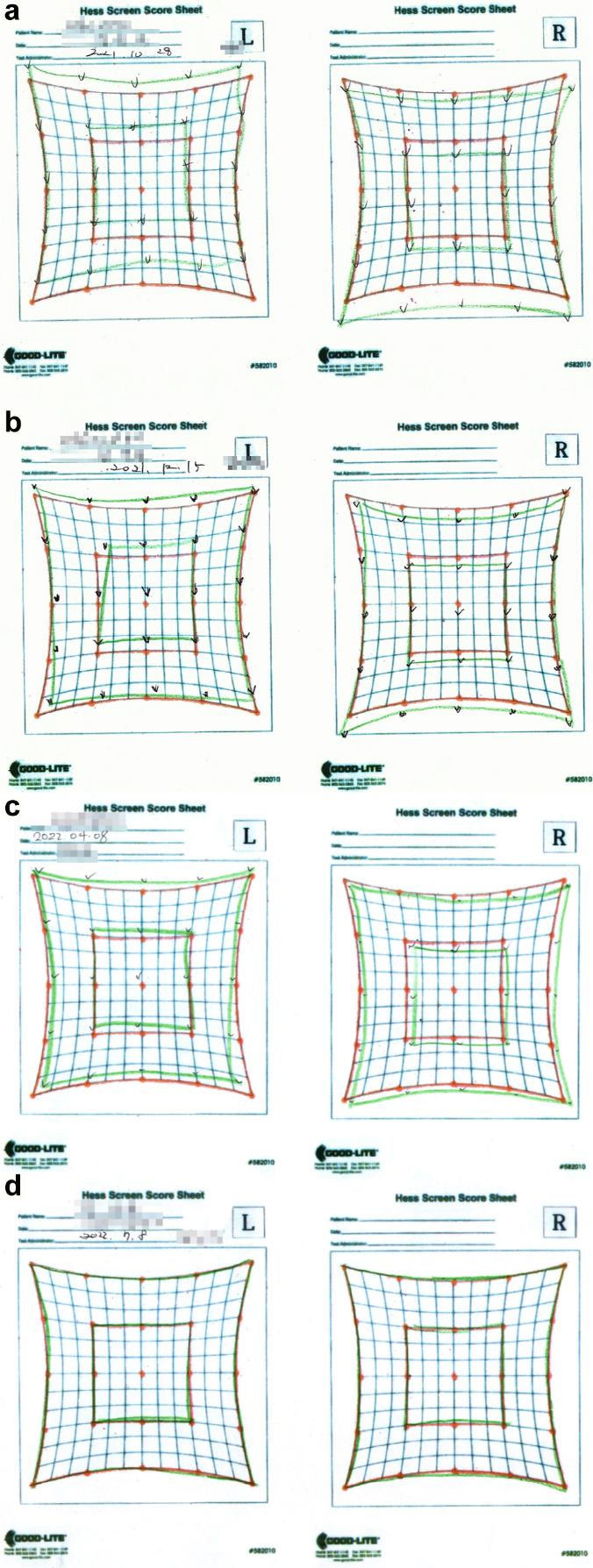


**Figure S1**. Hess screen tests in case 2, demonstrating left fourth nerve palsy. Hess screen test shows left fourth nerve palsy. (a) Week 5. (b) Week 12. (c) Week 28. (d) Week 41.


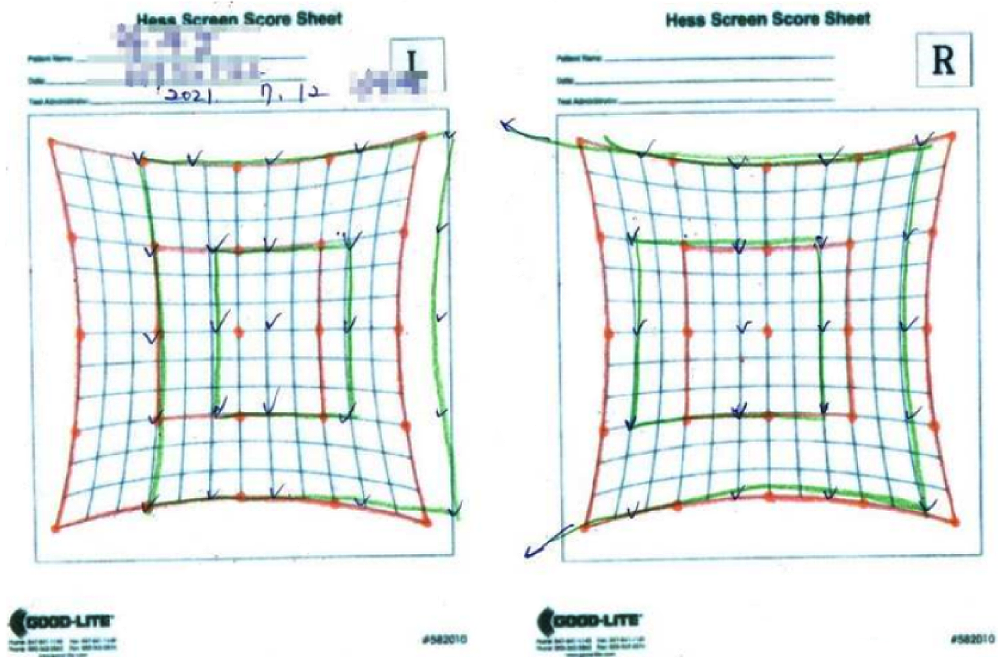


**Figure S2**. Hess screen test in case 3, demonstrating left sixth nerve palsy. Hess screen test shows left sixth nerve palsy.


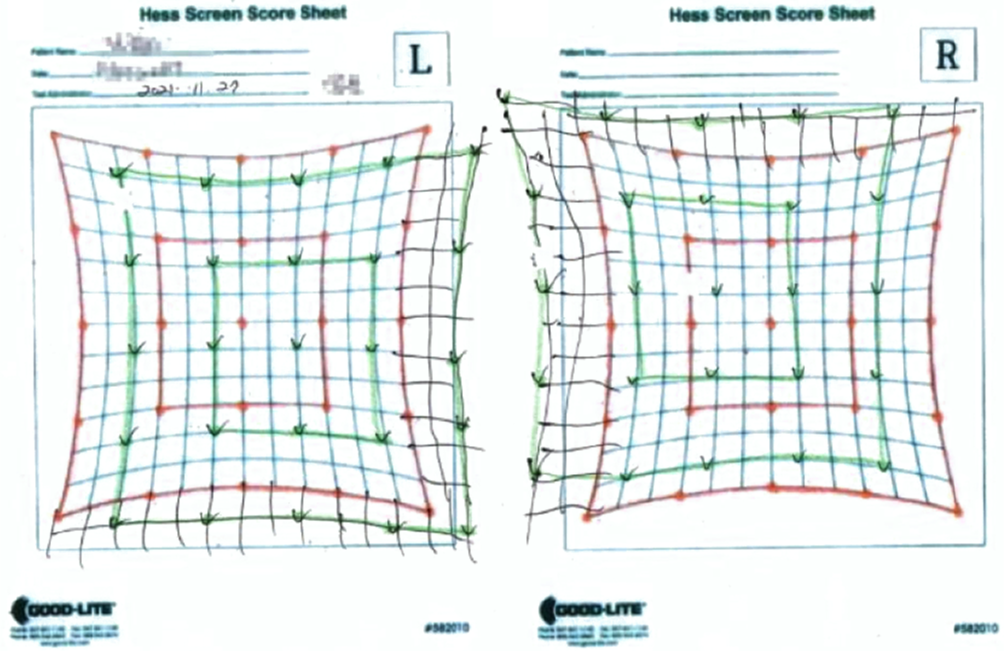


**Figure S3**. Hess screen test in case 4, demonstrating right sixth nerve palsy. Hess screen test shows right sixth nerve palsy.


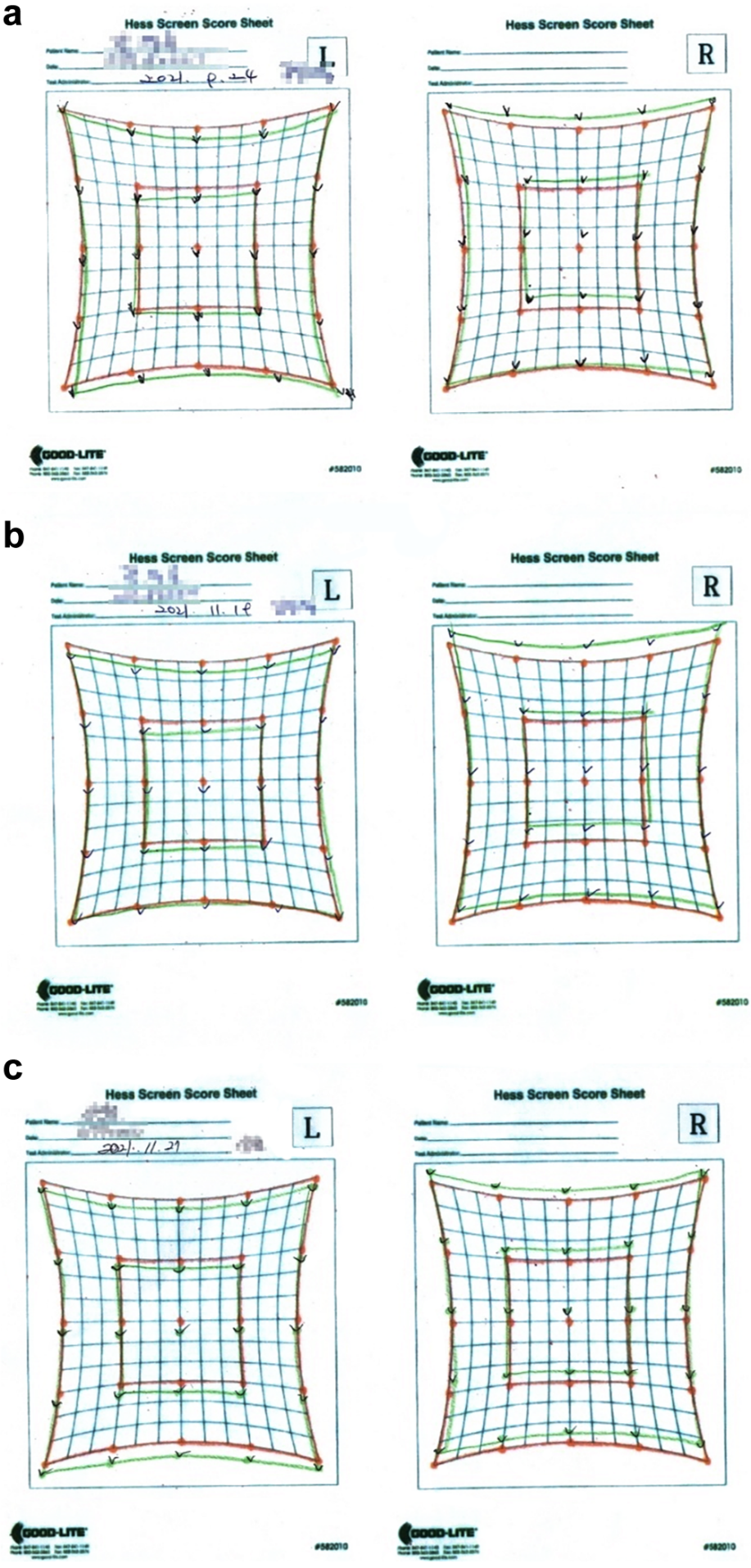


**Figure S4**. Hess screen tests in case 5, demonstrating right fourth nerve palsy. Hess screen test shows right fourth nerve palsy. (a) Week 16. (b) Week 25. (c) Week 27.


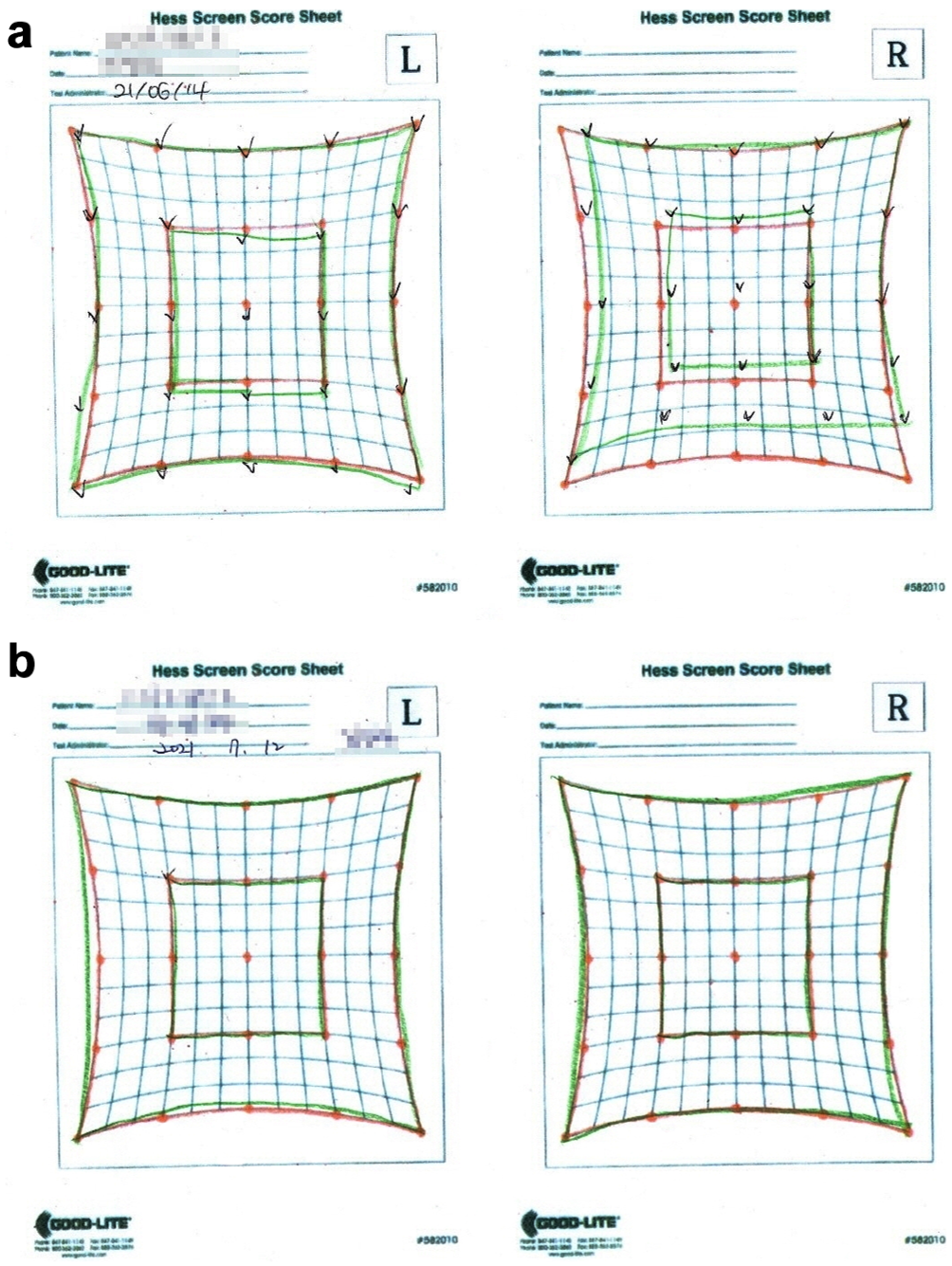


**Figure S5**. Hess screen test in case 7, demonstrating right fourth nerve palsy. Hess screen test shows right fourth nerve palsy. (a) Day 1. (b) Week 4.


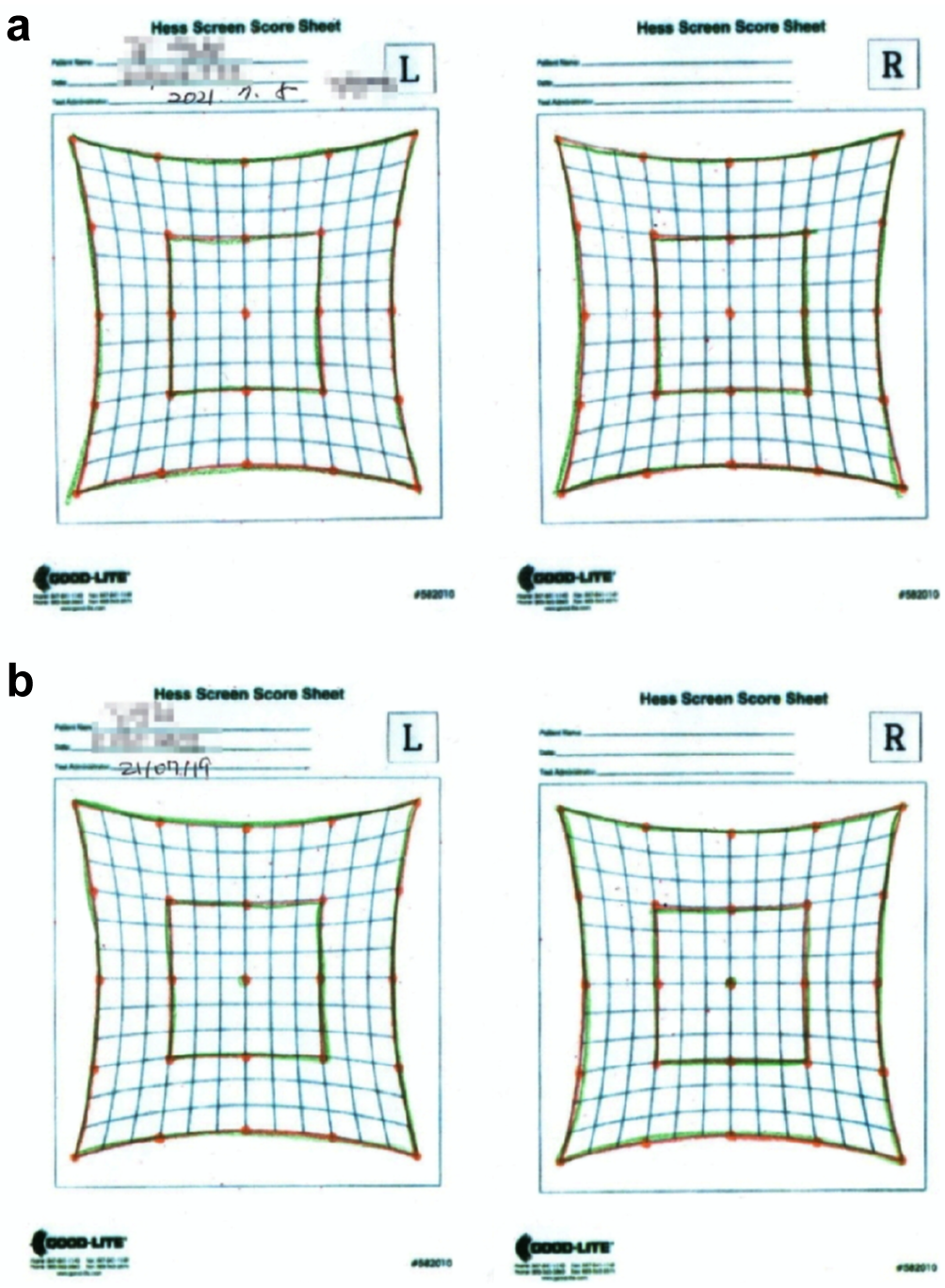


**Figure S6**. Hess screen test in case 9, demonstrating no eye movement defect. Hess screen test shows no eye movement defect. (a) Day 25. (b) Week 5.
